# Molecular Inversion Probe-Based Sequencing of *USH2A* Exons and Splice Sites as a Cost-Effective Screening Tool in USH2 and arRP Cases

**DOI:** 10.3390/ijms22126419

**Published:** 2021-06-15

**Authors:** Janine Reurink, Adrian Dockery, Dominika Oziębło, G. Jane Farrar, Monika Ołdak, Jacoline B. ten Brink, Arthur A. Bergen, Tuula Rinne, Helger G. Yntema, Ronald J. E. Pennings, L. Ingeborgh van den Born, Marco Aben, Jaap Oostrik, Hanka Venselaar, Astrid S. Plomp, M. Imran Khan, Erwin van Wijk, Frans P. M. Cremers, Susanne Roosing, Hannie Kremer

**Affiliations:** 1 Department of Human Genetics, Radboud University Medical Center, Geert Grooteplein Zuid 10, 6525 Nijmegen, The Netherlands; Janine.Reurink@radboudumc.nl (J.R.); Tuula.Rinne@radboudumc.nl (T.R.); Helger.IJntema@radboudumc.nl (H.G.Y.); Marco.Aben@wur.nl (M.A.); mimranmani@gmail.com (M.I.K.); Frans.Cremers@radboudumc.nl (F.P.M.C.); Susanne.Roosing@radboudumc.nl (S.R.); 2 Donders Institute for Brain Cognition and Behaviour, Radboud University Medical Center, 6500 Nijmegen, The Netherlands; Ronald.Pennings@radboudumc.nl (R.J.E.P.); Erwin.vanWyk@radboudumc.nl (E.v.W.); 3 The School of Genetics & Microbiology, Trinity College Dublin, D02 VF25 Dublin, Ireland; dockerya@tcd.ie (A.D.); Jane.Farrar@tcd.ie (G.J.F.); 4 Department of Genetics, Institute of Physiology and Pathology of Hearing, 02-042 Warsaw/Kajetany, Poland; d.ozieblo@ifps.org.pl (D.O.); m.oldak@ifps.org.pl (M.O.); 5 Postgraduate School of Molecular Medicine, Medical University of Warsaw, 02-091 Warsaw, Poland; 6 Department of Clinical Genetics, Amsterdam UMC, University of Amsterdam, 1105 Amsterdam, The Netherlands; j.b.tenbrink@amsterdamumc.nl (J.B.t.B.); aabergen@amsterdamumc.nl (A.A.B.); a.s.plomp@amsterdamumc.nl (A.S.P.); 7 Department of Ophthalmology, Amsterdam UMC, University of Amsterdam, 1105 Amsterdam, The Netherlands; 8 Department of Otorhinolaryngology, Radboud University Medical Center, 6500 Nijmegen, The Netherlands; Jaap.Oostrik@radboudumc.nl; 9 The Rotterdam Eye Hospital, 3000 Rotterdam, The Netherlands; born@oogziekenhuis.nl; 10 Centre for Molecular and Biomolecular Informatics, Radboud Institute for Molecular Life Sciences, Radboud University Medical Center, 6500 Nijmegen, The Netherlands; Hanka.Venselaar@radboudumc.nl

**Keywords:** *USH2A*, Usher syndrome type IIa, retinitis pigmentosa, molecular inversion probes (MIPs)

## Abstract

A substantial proportion of subjects with autosomal recessive retinitis pigmentosa (arRP) or Usher syndrome type II (USH2) lacks a genetic diagnosis due to incomplete *USH2A* screening in the early days of genetic testing. These cases lack eligibility for optimal genetic counseling and future therapy. *USH2A* defects are the most frequent cause of USH2 and are also causative in individuals with arRP. Therefore, *USH2A* is an important target for genetic screening. The aim of this study was to assess unscreened or incompletely screened and unexplained USH2 and arRP cases for (likely) pathogenic *USH2A* variants. Molecular inversion probe (MIP)-based sequencing was performed for the *USH2A* exons and their flanking regions, as well as published deep-intronic variants. This was done to identify single nucleotide variants (SNVs) and copy number variants (CNVs) in 29 unscreened or partially pre-screened USH2 and 11 partially pre-screened arRP subjects. In 29 out of these 40 cases, two (likely) pathogenic variants were successfully identified. Four of the identified SNVs and one CNV were novel. One previously identified synonymous variant was demonstrated to affect pre-mRNA splicing. In conclusion, genetic diagnoses were obtained for a majority of cases, which confirms that MIP-based sequencing is an effective screening tool for *USH2A*. Seven unexplained cases were selected for future analysis with whole genome sequencing.

## 1. Introduction

A substantial proportion of individuals with autosomal recessive retinitis pigmentosa (arRP) or Usher syndrome (USH) lacks a genetic diagnosis and therefore has reduced possibilities for future therapy and optimal genetic counseling. According to RetNet, variants in 63 and 16 genes have been associated with arRP and (atypical forms of) USH, respectively (RetNet consulted on 9 March 2021) [1]. *USH2A* defects are estimated to be causative of 8–19% of non-syndromic arRP cases [2,3], 29–55% of all USH cases and 57–90% of cases with USH type 2 (USH2) [4,5,6]. The prevalence of arRP and USH is estimated to range from 22–67 per 100,000 individuals [7,8] and from 4–6 per 100,000 individuals [9,10], respectively. This highlights the importance of screening the *USH2A* gene in medical genetic testing.

Screening of *USH2A* for defects can be challenging as the gene spans 800,503 nucleotides (nt) harboring 73 exons (NM_206933.2), including a cochlea-specific exon, exon 71 [11,12]. As a result of this expansive yet critical gene, large numbers of variants of unknown significance (VUS) have been detected, which adds to the complexity of identifying true causal variants. Furthermore, initial attempts to genetically screen *USH2A* were largely incomplete, as only the 21 initially identified exons were screened [13]. Another limitation of early screening endeavors was that the screening assessed only known pathogenic variants when using approaches such as the APEX-chip [14]. Although many USH2 and arRP cases were genetically solved by the aforementioned strategies, a substantial number of subjects did not receive an informative genetic diagnosis.

A genetic diagnosis is essential for informing and refining the prognosis of an individual’s genetic condition. It may also have an unquantifiable emotional impact on an individual’s wellbeing, as they may achieve confirmation regarding the cause of their condition. Moreover, it is essential to ascertain a genetic diagnosis to become eligible for any gene-specific therapy. Currently, important steps are being taken towards genetic therapy for *USH2A*-associated retinal disease. A phase 1/2 clinical trial is ongoing for evaluation of safety and tolerability of antisense oligonucleotides with the aim to induce skipping of exon 13 during pre-mRNA splicing (ClinGov ID: NCT03780257 (https://clinicaltrials.gov/ct2/show/NCT03780257 accessed on 15 February 2021), consulted on 15 February 2021). This is promising for many subjects as the most common pathogenic *USH2A* variants affect exon 13; c.2299del (p.(Glu767Serfs*21)) and c.2276G>T(p.(Cys759Phe)) [15]. A similar strategy has been proposed to prevent the inclusion of a frame-disrupting pseudo-exon (PE40) [16,17].

To address the potential disease association of *USH2A* in genetically unexplained USH2 and arRP cases, we envisaged a two-step strategy. The first step is screening the exons, intron-exon boundaries, and five regions of previously identified deep-intronic variants [16,18,19] with a fast and inexpensive molecular inversion probes (MIPs) approach. MIP-based sequencing is a next-generation sequencing method in which regions of 112 nucleotides are sequenced in multiplex [20,21]. The second step, a future endeavor, will be performing whole genome sequencing (WGS) to address variants in non-coding regions of the gene in the remaining monoallelic cases.

Here, we describe the efficacy of *USH2A* MIP-based sequencing for 40 unsolved arRP and USH2 cases. Fifty four percent of the arRP cases and 90% of the USH2 cases were genetically explained by *USH2A* variants. Four novel single nucleotide variants (SNVs) and one novel likely pathogenic exon deletion were identified. The pathogenic effect on splicing has also been demonstrated for one previously reported synonymous variant.

## 2. Results

Eleven arRP cases with monoallelic *USH2A* variants that met our criteria were included in the study. The previously identified variants in the DNA of these cases were five stop-gain variants and six missense variants (Table S1). Moreover, 29 genetically yet unexplained USH2 cases were included. DNA samples of 17 of the 29 subjects were (partially) pre-screened, whereby one *USH2A* variant was reported, while no prescreening was performed for the remaining 12 cases.

With MIP-based *USH2A* sequencing, on average 455,394 reads were obtained per sample. The coverage per MIP ranged from 0 to 21,685× with an average coverage of 1488× per sample.

### 2.1. One Hundred and Seven SNVs Were Identified, Including Four Novel Variants

After exclusion of variants based on coverage and allele frequency, 107 unique rare SNVs were identified, including all 28 variants that were reported at the start of the study (Table S1). All variants were classified according to the criteria mentioned in the materials and methods section. This resulted in 16 stop-gain and frameshift variants present in 36 alleles, three canonical splice site variants present in four alleles, 14 missense variants in 19 alleles, and one non-canonical splice site variant and two synonymous variants with a predicted effect on splicing. We identified one variant (c.12575G>A (p.(Arg4192His))) with conflicting interpretations in the Leiden Open (source) Variation Database (LOVD). However, this variant was classified as benign by a ClinVar expert panel (ClinVar consulted in March 2021) and was therefore excluded.

In addition, we detected three variants present in less than 15% of the reads that were previously reported in LOVD or ClinVar. One of these variants (c.11389+14del) is reported as ‘benign’ in LOVD, while a second variant (c.687C>A (p.(Gly229=))) was reported as ‘likely benign’ in ClinVar. A third variant (c.7067A>G (p.(Asn2356Ser))) had a classification of ‘likely pathogenic’ in ClinVar. This variant was, however, identified in a case (W19-0128) in which we identified already two known pathogenic variants (c.9258+1G>A and c.11864G>A (p.(Trp3955*))) and therefore not considered for further analysis. Sanger sequencing of the poorly covered regions in unsolved cases did not reveal any additional variants.

**Figure 1:**
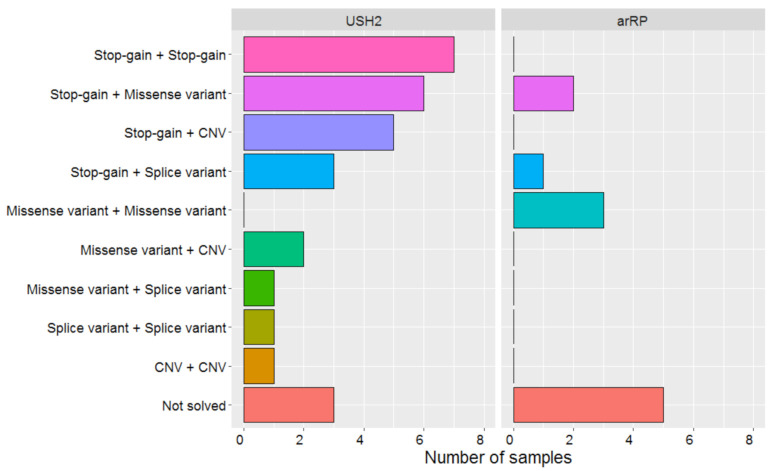
Overview of combinations of variant types identified in autosomal recessive retinitis pigmentosa (arRP) (*n* = 11) and Usher syndrome type 2 (USH2) (*n* = 29) cases. Splice variant: Canonical splice site variants and variants with any effect on splicing (both in frame and out-of-frame). CNV: Copy number variant.

Four of the identified SNVs are novel (Table 1); two of these are predicted to result in a stop-gain and are classified as pathogenic, two variants were missense variants that we classified as likely pathogenic. An overview of the combinations of types of variants that were deemed (likely or potentially) pathogenic in both subject groups is depicted in Figure 1.

After assessment of all rare missense, synonymous and intronic variants, SpliceAI was employed to predict an effect on pre-mRNA splicing for any of the identified SNVs. Two of the identified intronic variants (c.7595−3C>G and c.14583−20C>G) were predicted to affect their nearby canonical splice sites, which was experimentally confirmed previously for c.7595−3C>G (p.(Pro2533Asnfs*5)) [22]. Two synonymous variants (c.949C>A and c.8709C>T) were predicted to affect splicing. For the former variant, Vaché et al. [23] demonstrated the effect on the protein to be p.(=,Tyr318Cysfs*17). As expected, the three detected canonical splice site variants (c.9258+1G>A, c.9371+1G>C and c.11048−2A>G) were predicted to affect splicing and these are considered pathogenic in LOVD or ClinVar.

**Figure 2:**
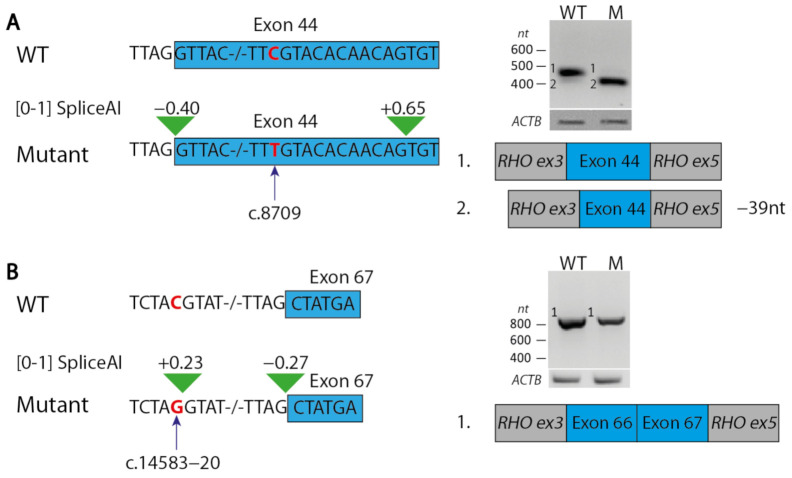
Results of minigene splice assays for variants c.8709C>T and c.14583−20C>G. (**A**) Variant c.8709C>T (indicated in red) is predicted by splice prediction tool SpliceAI to result in a 39-nt deletion of exon 44 in the *USH2A* transcript. This effect was indeed observed in a splice assay for the construct containing the variant (M), but not for the wildtype construct (WT). (**B**) SpliceAI predicts an increased strength of a non-canonical splice donor site due to variant c.14583−20C>G. A splice assay did not demonstrate the use of this splice site as no elongation of exon 67 was observed. Exonic sequences are boxed (blue). Green triangles indicate SpliceAI predictions.

We performed minigene splice assays for variants c.14583−20C>G and c.8709C>T (Figure 2). Variant c.8709C>T was predicted to create a cryptic splice acceptor site within exon 44, 12 nucleotides downstream of the variant (SpliceAI score: 0.65/1), and to result in the loss of the canonical splice acceptor site (SpliceAI: 0.4/1). Indeed, an in-frame deletion of 39 nt of exon 44 was observed, which encodes part of a fibronectin type III (FN3) domain, while an insubstantial fragment was observed representing the wildtype exon 44. Variant c.14583−20C>G was predicted to create a cryptic splice acceptor site located 19 nt upstream of the canonical splice acceptor site of exon 67 (SpliceAI: 0.27/1). However, the splice assay did not provide any evidence that this variant had an effect on *USH2A* pre-mRNA splicing. Therefore, we classified this variant as VUS.

### 2.2. Four Copy Number Variants Were Detected

In this study, three previously reported and one novel copy number variants (CNVs) were detected in nine alleles (Table 2); for two of these CNVs, the breakpoints were exactly as reported in LOVD (c.1644+10004_1972−12164del [24] and c.4627+25435_4987+660del [25,26]). The c.8559-?8681+?del (p.(Tyr2854_Arg2894del)) CNV is novel and is predicted to result in the in-frame loss of exon 43 (123 nt). We were not able to determine the breakpoints of this CNV with PCR. A fourth CNV is potentially identical to the deletion reported by Zampaglione et al. [27], who did not assess the breakpoints. We characterized the CNV as an in-frame deletion of exons 38-56 with a 12-nt insertion at the breakpoint (c.7121−8313_11048−962delins12 (p.(Val2374_Gly3683del))). The deletion is predicted to result in the (partial) loss of 12 fibronectin type-III domains of usherin, and this CNV was classified as pathogenic. We identified this deletion in USH2 cases from five families in our cohort, and variable-number tandem repeat (VNTR) marker analysis in four out of five families revealed a shared haplotype (Figure S1). 

### 2.3. Two In-Frame Deletions Were Classified as Likely Pathogenic after Modelling

Variants c.8709C>T (p.(Arg2894_Asn2906del,=)) and c.8559-?_8681+?del (p.(Tyr2854_Arg2894del)) result in an in-frame deletion of 13 and 41 amino acids, respectively. While variant c.8559−? 8681+?del is not reported in LOVD or ClinVar, variant c.8709C>T is only classified as a synonymous variant without considering its effect on splicing. The homology model of the affected FN3 domain indicated that both in-frame deletions will be detrimental to the protein structure. The deletion of 13 amino acids is predicted to result in the deletion of a full beta-strand (Figure S2A). Significant remodeling of this domain will be necessary to connect the remaining amino acids. The variant that results in the deletion of 41 amino acids even leads to the deletion of approximately half of the domain structure (Figure S2B). In both cases, a large structural rearrangement can be expected to accommodate the deletion of 13 and 41 amino acids. This will severely affect the protein structure and therefore both variants were labeled pathogenic.

In total, 32 out of 40 cases were considered genetically explained with bi-allelic *USH2A* variants after MIP-based sequencing and in-depth data analysis. One USH2 sample was genetically explained with a homozygous missense variant (c.4108T>G) in *ADGRV1* through a parallel screening effort. All potentially pathogenic, likely pathogenic and pathogenic variants identified in this study are listed per subject in Table S1.

## 3. Discussion

With our strategy for *USH2A* screening with MIPs, 32 out of 40 cases received a genetic diagnosis as two (likely or potentially) pathogenic variants were identified. In three of these cases a (likely) pathogenic variant was detected in combination with a missense VUS that met our pathogenicity criteria. The overall solve rate of our method was 80%. If only USH2 cases are considered, the solve rate was 26 out of 29 (90%).

Five of the variants identified in our study are novel: two stop-gain variants, two missense variants (Table 1) and a CNV (Table 2). The novel CNV c.8559-?_8681+?del (p.(Tyr2854_Arg2894del) and the known synonymous variant c.8709C>T (p.(Arg2894_Asn2906del,=) with an effect on splicing were predicted and likely to result in an in-frame shortening of the *USH2A* transcript. Due to their in-frame effect, pathogenicity of these variants was not obvious. We employed in silico modelling for both variants to increase support for a possible pathogenic effect. For the deletion of exon 43, modeling predicts a severe effect on the structure of fibronectin type-III domain 15 (Figure S2B). The combination of this deletion with a stop-gain (c.11864G>A; p.(Trp3955*)) *in trans* that was observed in this case supports the classification of the deletion of *USH2A* exon 43 as pathogenic. As we were not able to identify the exact breakpoints for this deletion with PCR, we hypothesize that this CNV may be the result of a more complex event, such as the combination of a deletion and an inversion.

Variant c.8709C>T resulted in a nearly complete effect on splicing that leads to a 13 amino acid deletion in usherin (p.(Arg2894_Asn2906del,=). This variant results in reduction of fibronectin type-III domain 15 and is suggested to have a severe effect on domain structure (Figure S2A). As parental DNA was not available for segregation and these variants have not been reported in combination before, we assume that the c.8709C>T variant and variant c.3187_3188del (p.(Gln1063Serfs*15)) are in trans and are likely to explain the phenotype. For variant c.14583−20C>G, no effect on splicing was observed in the minigene splice assay that was performed in HEK293T-cells. Previous research pointed out that splice defects can be retina-specific [29] and testing this variant in iPSC-derived photoreceptor precursor cells could be an interesting next step to observe a possible effect on splicing.

The identification of private *USH2A* variants is consistent with previous studies [24,30]. Nevertheless, we also identified a recurrent variant, the deletion c.7121−8313_11048−962delins12 (p.(Val2374_Gly3683del)) in six alleles. Haplotype sharing of the alleles with the deletion and the descent of the subjects suggest that this deletion is a Dutch founder mutation. This deletion was not present in the Genome Aggregation Database (gnomAD) SV database (10,847 genomes) and the 1000 Genomes database [31,32]. This suggests that the deletion c.7121−8313_11048−962delins12 is absent or very rare in the populations represented in these datasets (African/African American, Latino, East Asian, South Asian and European). Also in the Dutch population, this variant is very rare as it is not present in our in-house WGS database (780 unrelated individuals).

Another recurrent variant, c.11864G>A (p.(Trp3955*)), was present as the first allele in 11 of the cases screened with *USH2A* MIP-based sequencing. Ten of these cases are of Polish descent which were only pre-screened for this variant prior to inclusion in this study. Previously, this variant has been reported to be recurrent in several populations, and specifically in the Central-Eastern European population with frequencies up to 82.5% in a cohort of Slovenian *USH2A*-associated USH2 cases [30,33].

Although cohorts among different studies are difficult to compare due to variable inclusion criteria, extent of prescreening and cohort size, we achieved a yield for USH2 that is comparable to that reported in literature. A solve rate of 89% was indicated for *USH2A* screening in Scandinavia using Sanger sequencing [34] and Bonnet and coworkers identified two (likely) pathogenic *USH2A* variants in 88.4% of 258 USH2 cases through targeted exome sequencing, SNP arrays and qPCR [30]. 

Eight cases from our cohort remained genetically unexplained after screening with *USH2A* MIPs. One of these was an USH2 case, who was diagnosed with a homozygous missense variant in *ADGRV1* in a parallel study. For the seven remaining cases, WGS will be performed as the second step of our approach to identify novel deep-intronic variants, structural variants such as inversions, and variants in regulatory regions of *USH2A*. Besides variants that cannot be detected through our current design for *USH2A* MIP-based sequencing, pathogenic variants in other genes associated with USH2 might also explain unsolved USH2 cases, as we demonstrated for one of the studied subjects. Similarly, in a study by Bonnet et al., 7% of the USH2 cases had two variants in *ADGRV1* and in a recent meta-analysis it was estimated that ~10% of USH2 cases can be explained by variants in *ADGRV1* and *WHRN* [4,30]. In addition, bi-allelic variants in genes associated with USH type I have been reported in subjects phenotypically classified as USH2 [30], demonstrating that genotype-phenotype correlations for the three USH types are not completely distinct. Finally, a rare alternative explanation for the missing heritability is that subjects diagnosed with USH suffer from a concurrence of non-syndromic hereditary hearing loss and arRP. For non-syndromic arRP, the probability of being a carrier of an *USH2A* variant in combination with bi-allelic pathogenic variants in a different gene is higher than for USH due to its enormous genetic heterogeneity; 83 genes are currently associated with non-syndromic arRP and the closely related cone-rod dystrophy [1]. 

Our study demonstrates that *USH2A* MIP-based sequencing is a valuable pre-screening tool for unscreened or partially screened cases with a high probability of carrying pathogenic *USH2A* variants. We calculated that *USH2A* MIP-based sequencing will cost ~€15 per sample for reagents only (excluding MIPs design and synthesis) when a batch of samples (>150) is screened in parallel. As USH2 is not very heterogeneous and the phenotype is distinct, this method can be competitive compared to WES (~€175) in screening large numbers of unscreened or partially screened USH2 cases, as well as partially screened mono-allelic arRP cases. Furthermore, MIP-based sequencing is fast and enables detection of known deep-intronic variants. It is also flexible and MIPs could be included for other genes associated with USH and arRP. In contrast, WES and WGS allow for the simultaneous screening for variants throughout the exome/genome which is beneficial in cases for which phenotypic clues are limited or ambiguous. With WGS, one can detect novel deep-intronic and intergenic variants and more complex CNVs and structural variants. Although WGS is being broadly implemented in medical genetic testing, it is still relatively expensive. 

In conclusion, MIP-based sequencing of *USH2A* is a cost-effective method for genetic screening of USH2 cases and arRP cases with monoallelic *USH2A* variants. As there is a high likelihood that these cases carry bi-allelic variants in *USH2A*, it is very efficient to focus screening on the coding region and known deep-intronic variants of this gene as a first step, prior to WGS. With our strategy we were able to identify five novel *USH2A* variants and provide 80% of the included subjects with a genetic diagnosis.

## 4. Materials and Methods

### 4.1. Inclusion of Samples

DNA of genetically unexplained subjects was obtained from centers in the Netherlands, Ireland and Poland. Written informed consent was received from all individuals, adherent to the tenets of the Declaration of Helsinki and as approved by the local ethics committee of each participating center (NL33648.091.10, NL34152.078.10, 2020604, KB/273/2012). Cases with a typical USH2 or arRP phenotype were included. As the *USH2A* gene is involved in 57–90% of all USH2 cases, but only in 8–19% of arRP cases, inclusion criteria differed between USH2 cases and arRP cases. For subjects with USH2, the inclusion criterion was that no screening, or only partial pre-screening of the *USH2A* coding region had been performed. Cases of arRP were included if they were partially pre-screened and one variant had previously been identified in *USH2A* meeting the following criteria; being (1) a stop-gain variant, (2) a frameshift variant, (3) a canonical splice site variant, or (4) a missense variant which either has a classification of VUS, likely pathogenic, or pathogenic according to LOVD. Furthermore, arRP cases were included with monoallelic *USH2A* variants that have a minor allele frequency (MAF) ≤1% in gnomAD (GRCh37) [35]. In addition, these missense variants had to meet at least four out of five criteria: (i) a Grantham score ≥80 (range: 5–215) [36], (ii) a CADD_PHRED score ≥15 (range: 0–99) [37], (iii) a PhyloP ≥2.7 [38], (iv) a SIFT label of ‘Deleterious’ [39] and (v) a MutationTaster label of ‘Disease causing’ [40].

### 4.2. MIP-Based Sequencing and Data Analysis

Three hundred and twenty-one MIPs were designed to cover all coding regions of *USH2A* (NM_206933.2), at least 20 nt of intronic sequences upstream and downstream of all exons and four currently known deep-intronic variants (c.5573−843A>G, c.7595−2144A>G, c.8845+628C>T and c.9959−4159A>G) [16,18]. A more recently identified fifth deep-intronic variant (c.14134−3169A>G) was analyzed by Sanger sequencing [19]. MIPs were designed with the use of an in-house pipeline based upon the script and guidelines provided by the Shendure Laboratory (University of Washington, Seattle, WA, USA) [21]. Probes were added to genomic DNA of the selected cases for amplification and inclusion of an 8-nt barcode to each amplicon. Samples were pooled, purified and sequenced on an Illumina NextSeq 500 system. Alignment and processing of data was performed using BWA mem and an in-house analysis and variant interpretation pipeline [41]. GATK was used to call the SNVs and small indels [42]. A more elaborate description of this pipeline is provided in the supplementary methods.

Variants represented by less than 15% of the reads were only considered if they were reported in LOVD or ClinVar. Variants at the last nucleotide of a MIP which were not present in an overlapping MIP were removed as these represent a known artifact and these variants were unlikely to be present. For unsolved cases, the BAM files were manually checked to identify candidate variants in poorly covered regions (<10 reads). Regions with <5 reads and poorly covered regions in which the presence of rare variants was suggested were assessed by PCR and subsequent Sanger sequencing.

### 4.3. Prioritization and Classification of Variants

All SNVs with a MAF ≤1% were evaluated and classified according to the aforementioned criteria and guidelines of the American College of Medical Genetics and Genomics (ACMG) [43]. To identify variants with a potential effect on splicing, we assessed splice predictions for all SNVs using SpliceAI [44]. Variants with a predicted delta score of ≥0.2 (range: 0–1) for at least two of the four predictions (acceptor gain, acceptor loss, donor gain, donor loss) were considered for a minigene splice assay. Stop-gain variants, canonical splice site variants and variants in cis with a stop-gain variant were excluded from a splice assay.

CNVs were evaluated in all samples, as described previously [45]. If seven consecutive MIPs had a normalized coverage ≤0.7 a deletion was suspected, while a duplication was assumed if seven consecutive MIPs had a normalized coverage ≥1.3. Genomic qPCR analysis was performed according to standard protocols if the normalized coverage of fewer than seven MIPs suggested a CNV. For all detected CNVs, a breakpoint PCR was performed (primers sequences provided in Table S2), followed by Sanger sequencing. A VNTR marker analysis was performed according to standard protocols to detect a shared haplotype encompassing a deletion from exon 38 to exon 56 by testing the VNTR markers D1S22629, D1S2827 and D1S229. For all variants, segregation analysis was performed using Sanger sequencing if DNA of family members was available. All pathogenic and likely pathogenic variants identified in this study have been submitted to LOVD (www.lovd.nl/USH2A (accessed on 15 February 2021)).

### 4.4. Minigene Splice Assays

To perform minigene splice assays, the affected *USH2A* exons and at least 1 kb of up- and downstream intronic sequence were amplified from genomic DNA derived from the individual carrying the variant of interest (primer sequences and the sizes of the amplicons are listed in Table S3). Both the wildtype fragments and fragments containing the variants were individually cloned in an adapted pCI-*NEO* vector [46], with Gateway^®^ cloning technology (Thermo Fisher Scientific, Carlsbad, CA, USA) between *RHO* exons 3 and 5. The inserts were verified by Sanger sequencing. Wildtype and mutant minigene splice vectors (500 ng) were subsequently transfected individually into HEK293T-cells using polyethylenimine (100 µg/mL in 150 mM NaCl). Approximately 24 h post-transfection, RNA was isolated by employing the NucleoSpin RNA Clean-up kit (Macherey-Nagel, Düren, Germany). One microgram of RNA was used for cDNA synthesis using the iScript cDNA Synthesis kit (Bio-Rad, Hercules, CA, USA) following the manufacturer’s instructions. RT-PCR was performed with primers for *RHO* exons 3 and 5 to observe the splicing pattern in the *USH2A*-regions of interest. A PCR for *ACTB* was performed as a lysis control. PCR products were size-separated on a 1% agarose gel and bands were excised from gel and purified using the NucleoSpin Gel & PCR Clean-up kit (Machery Nagel, Düren, Germany) prior to Sanger sequencing.

### 4.5. Modelling the Effect of Two In-Frame Deletions

As a crystallized structure for the complete usherin protein has not yet been resolved and two identified in-frame deletions affect the 15th fibronectin-3 domain, a homology model was created spanning amino acids 2820–2923 (NM_206933.2).

The homology modelling experiment was performed using the WHAT IF & YASARA Twinset automatic modelling module [47,48]. Several models were created and combined into a single best-fit hybrid model, predominantly informed by PDB file 4U3H [49]. Small parts of models created with PDB files 4YFE, 6TPW and 4M4P were also used [50,51,52]. The final hybrid model was subjected to one more energy minimization round and used for further analysis. The usherin sequence and the templates share between 25-35% sequence identity. This indicates that the model can provide a good overall impression of the 3D-conformation, but the information regarding the atomic structure is limited.

## Figures and Tables

**Table 1:** Novel SNVs identified in this study.

Variant (NM_206933.2)	Protein Effect	Exon	PhyloP (≥2.7)	CADD_ PHRED (≥15)	Grantham (≥80)	SIFT	Mutation Taster	Domain	ACMG Classification
c.9388T>G	p.(Trp3130Gly)	48	5.06	25.5	184	Deleterious	Disease causing	Fibronectin type-III domain 18	Likely Pathogenic
c.11683G>T	p.(Gly3895*)	60	5.89	55	-	-	-	Fibronectin type-III domain 23	Pathogenic
c.14303A>C	p.(Tyr4768Ser)	65	4.17	26.1	144	Deleterious	Disease causing	Fibronectin type-III domain 32	Likely Pathogenic
c.15286del	p.(Glu5096Lysfs*6)	70	-	41	-	-	-	-	Pathogenic

All variants are absent from the gnomAD population database. All novel single nucleotide variants (SNVs) were identified in one proband each; all of these probands were Usher syndrome type 2 (USH2) cases. *USH2A* protein domains were determined with the SMART protein domain annotation tool. ACMG: American College of Medical Genetics and Genomics.

**Table 2:** Overview of CNVs identified in this study.

Variant (NM_206933.2)	Protein Effect	Exons	Domains (Partially) Deleted	Number of Alleles	Reference
c.1644+10004_1972−12164del	p.(Cys549_Gln657del)	10-11	Laminin EGF-like domains 1-3	1	[24]
c.4627+25435_4987+660del	p.(Gly1543_Pro1662del)	22-24	Laminin G domain 1	1	[25,26]
c.7121−8313_11048−962delins12	p.(Val2374_Gly3683del)	38-56	Fibronectin type-III domains 10-21	6	[27]
c.8559-?_8681+?del	p.(Tyr2854_Arg2894del)	43	Fibronectin type-III domain 15	1	This study

*USH2A* protein domains were determined with the SMART protein domain annotation tool [28].

## Data Availability

All pathogenic and likely pathogenic variants identified in this study have been submitted to LOVD (www.lovd.nl/USH2A accessed on 15 February 2021).

## Supplementary Materials

The following are available online at https://www.mdpi.com/article/10.3390/ijms22126419/s1, Supplementary methods: An elaborate description of the MIP-based sequencing pipeline. Figure S1: Genotypes of VNTR-markers in four families carrying c.7121-8313_11048−962delins12. Figure S2: In silico modeling of the effect of c.8709C>T(p.(Arg2894_Asn2906del,=)) and c.8559−?_8681+?del(p.(Tyr2854_Arg2894del)). Table S1: All variants identified in this study that were classified as (likely or potentially) pathogenic. Table S2: Sequences of primers employed to determine the CNV breakpoints that were identified in this study. Table S3: Sequences of primers that were employed to create the constructs for the minigene splice assays.
