# Supplementary material for: Molecular Inversion Probe-Based Sequencing of USH2A Exons and Splice Sites as a Cost-Effective Screening Tool in USH2 and arRP Cases
Source: Int J Mol Sci. 2021 Jun 15;22(12):6419. doi: 10.3390/ijms22126419 (PMC8232728; doi:10.3390/ijms22126419)
Supplement: Supplementary file 1 [file ijms-22-06419-s001.zip › Supplementary data/Figure S1.pdf]

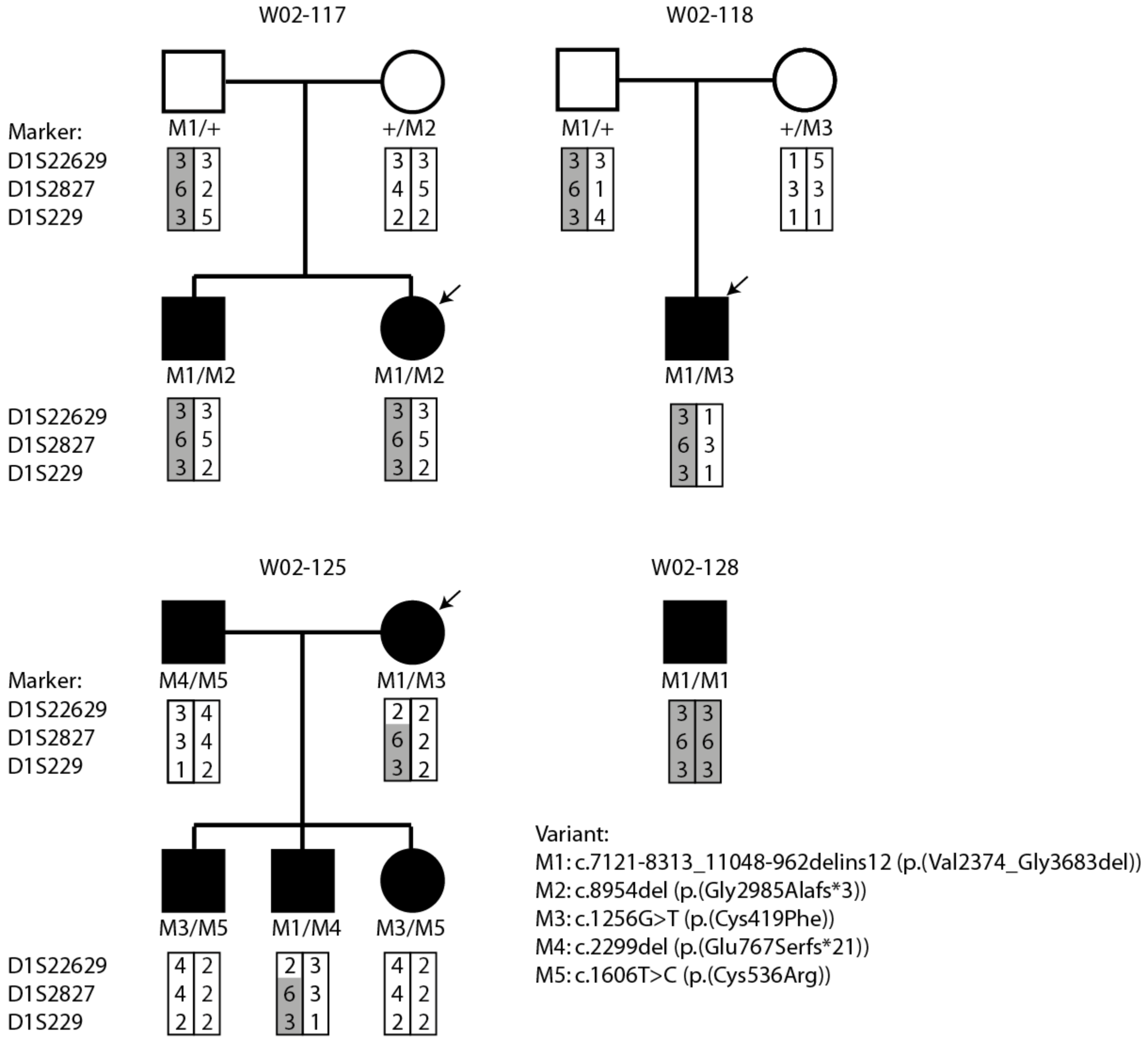

Figure S1: Genotypes of VNTR-markers in four families carrying c.7121-8313_11048-962delins12. The haplotype with the deletion is highlighted in grey. Probands of complex families that were included in our study are indicated with an arrow.
