# Supplementary material for: Molecular Inversion Probe-Based Sequencing of USH2A Exons and Splice Sites as a Cost-Effective Screening Tool in USH2 and arRP Cases
Source: Int J Mol Sci. 2021 Jun 15;22(12):6419. doi: 10.3390/ijms22126419 (PMC8232728; doi:10.3390/ijms22126419)
Supplement: Supplementary file 1 [file ijms-22-06419-s001.zip › Supplementary data/Figure S2.pdf]

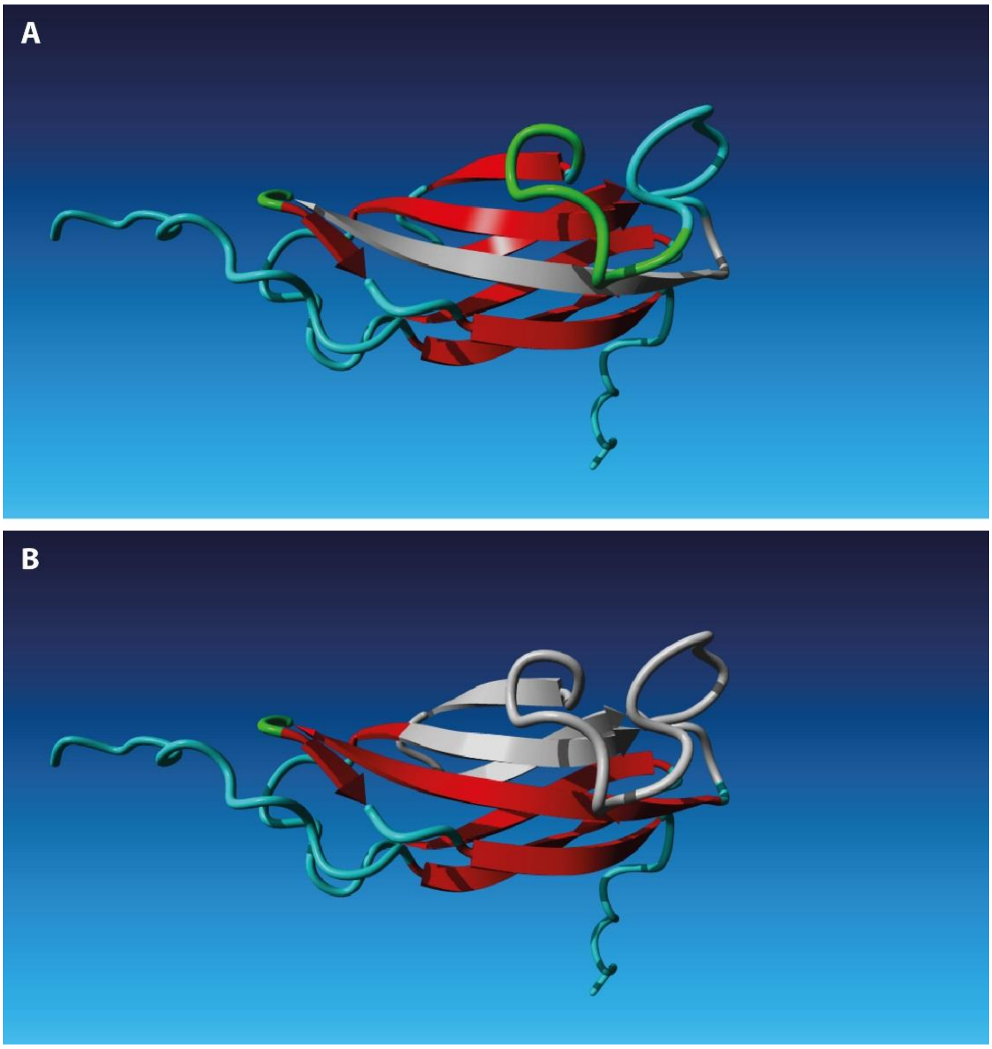

Figure S2: In silico modeling of the effect of (A) c.8709C>T (p.(Arg2894_Asn2906del,=)) and (B) c.8559-?_8681+?del (p.(Tyr2854_Arg2894del)). The models are colored by secondary structure element (red = beta-strand, green=turn, cyan=loop). The deleted protein regions are depicted in grey.
