# Supplementary material for: Molecular Inversion Probe-Based Sequencing of USH2A Exons and Splice Sites as a Cost-Effective Screening Tool in USH2 and arRP Cases
Source: Int J Mol Sci. 2021 Jun 15;22(12):6419. doi: 10.3390/ijms22126419 (PMC8232728; doi:10.3390/ijms22126419)
Supplement: Supplementary file 1 [file ijms-22-06419-s001.zip › Supplementary data/Supplementary methods.pdf]

Supplementary methods

MIPs were designed by employing an in-house MIP synthesis pipeline based upon the script and guidelines provided by Shendure Lab (the University of Washington, Seattle, USA) (http://krishna.gs.washington.edu/mip_pipeline). The specific arms of each probe target a 112-bp genomic region. These genomic targets were captured and processed according to the protocol provided by O'Roak BJ et al. [21], with few modifications. The individual genomic target was captured by a polymerization and gap filling reaction that results in a circular molecule of 182 bp. From the individual MIP stocks, synthesized as 70-mer by Integrated DNA Technologies (IDT), equal amounts were pooled to make a grand pool. The pool was then phosphorylated using 100 units of T4 polynucleotide kinase (NEB, MA, USA), at 37°C for 45 minutes. The phosphorylated MIPs pool combined with 10 units Hemo Klentaq (NEB, MA, USA, M0332S), 1 unit Ampligase (Epibio, A0102K) and 0.25mM dNTPS were used to capture the genomic targets at 60°C for 24 hours after an initial denaturation at 95°C for 10 minutes, in a final volume of 25 µl. 100 ng (5 µl) genomic DNA was used for each capture reaction. After 24 hours the capture reaction was stopped by adding 5 units of EXO I (NEB, M0293L) & EXO III (NEB, M0206L) mix, followed by an incubation at 37°C for 45 minutes, and 95°C for 2 minutes. Using 5 µl of EXO-treated capture and 1X iProof™ high-fidelity Master Mix (Bio-Rad, CA, USA), a PCR, was performed with primers corresponding to the common linker of the MIPs to append Illumina sequence adaptors. The reverse primer contained in addition to the sequence adaptors the 8-bp sample-specific barcode sequence. PCR was performed with an initial denaturation step at 98°C for 30 seconds, 18 cycles each of 98°C for 10 seconds, 60°C for 30 seconds, 72°C for 30 seconds, and a final extension at 72°C for 2 minutes. To remove primer dimer and empty MIP probes, equal amounts of PCR from each individual sample were pooled and purified with 1X AMPure XP beads (Beckman Coulter, CA, USA), according to standard protocol. Purification was validated with the

Agilent2200 TapeStation system (Agilent, CA, USA). The pool was quantified using a Qubit® fluorometer (ThermoFisher, MA, USA), and 4nmol of the library was sequenced on a Nextseq500 (Illumina), using the high output 2 x 75 bp kit, according to manufacturer instructions.

The 150 bp paired end reads were mapped to the reference human genome (GRCh37/hg19 assembly) using an in-house bioinformatics MIP-pipeline. The alignment was performed using BWA mem (v0.7.12) [41]. Processing of data was performed with an in-house analysis and variant interpretation pipeline. GATK (v3.4-46) was used to call the SNVs and small indels [42]. Each sample was called twice, using the HaplotypeCaller and the UnifiedGenotyper. After the variant calling, the variants of all samples were merged into two multi-VCFs using the GATK joint genotyping step for the HaplotypeCaller samples and the GATK CombineVariants step for the UnifiedGenotyper samples. Both multi-VCFs were annotated using the in-house annotation pipeline.
