# Supplementary material for: Molecular Inversion Probe-Based Sequencing of USH2A Exons and Splice Sites as a Cost-Effective Screening Tool in USH2 and arRP Cases
Source: Int J Mol Sci. 2021 Jun 15;22(12):6419. doi: 10.3390/ijms22126419 (PMC8232728; doi:10.3390/ijms22126419)
Supplement: Supplementary file 1 [file ijms-22-06419-s001.zip › Supplementary data/Table S1.pdf]

Table S1: All variants identified in this study that were classified as (likely or potentially) pathogenic.

Family ID	Phenotype	Variants (NM_206933.2)	Protein effect variants	ACMG classication	Variants segregate with disease?	Remark
W02-117	USH2	c.8954del	p.(Gly2985Alafs*3)	Pathogenic	Yes	
		c.7121−8313_11048−962delins12	p.(Val2374_Gly3683del)	Pathogenic		
W02-118	USH2	c.8079G>A	p.(Trp2693*)	Pathogenic	Yes	
		c.7121−8313_11048−962delins12	p.(Val2374_Gly3683del)	Pathogenic		
W02-125	USH2	c.1256G>T	p.(Cys419Phe)	Pathogenic	Yes	
		c.7121−8313_11048−962delins12	p.(Val2374_Gly3683del)	Pathogenic		
W02-128	USH2	c.7121−8313_11048−962delins12	p.(Val2374_Gly3683del)	Pathogenic	NT	
		c.7121−8313_11048−962delins12	p.(Val2374_Gly3683del)	Pathogenic		
W02-130	USH2	c.10561T>C	p.(Trp3521Arg)	Pathogenic	NT	
		c.949C>A	p.(=,Tyr318Cysfs*17)	Pathogenic		
W02-142	USH2	c.2299del	p.(Glu767Serfs*21)	Pathogenic	NT	
		c.14384T>G	p.(Leu4795Arg)	Likely Pathogenic		
W02-440	USH2	c.6722C>T	p.(Pro2241Leu)	Likely Pathogenic	NT	
		c.7121−8313_11048−962delins12	p.(Val2374_Gly3683del)	Pathogenic		
W03-377B	USH2				NT	Solved with variants in *ADGRV1*
W09-0017	USH2	c.2299del	p.(Glu767Serfs*21)	Pathogenic	NT	WGS initiated
W19-0114	arRP	c.14384T>G	p.(Leu4795Arg)	Likely Pathogenic	NT	
		c.13274C>T	p.(Thr4425Met)	Likely Pathogenic		
W19-0115	arRP	c.1227G>A	p.(Trp409*)	Pathogenic	NT	
		c.14384T>G	p.(Leu4795Arg)	Likely Pathogenic		
W19-0117	arRP	c.2276G>T	p.(Cys759Phe)	Pathogenic	NT	WGS initiated

W19-0118	arRP	c.2299del	p.(Glu767Serfs*21)	Pathogenic	NT	WGS initiated
W19-0120	arRP	c.9815C>T	p.(Pro3272Leu)	Likely Pathogenic	NT	
		c.9433C>T	p.(Leu3145Phe)	VUS		
W19-0121	arRP	c.2276G>T	p.(Cys759Phe)	Pathogenic	NT	
		c.1227G>A	p.(Trp409*)	Pathogenic		
W19-0123	arRP	c.2276G>T	p.(Cys759Phe)	Pathogenic	NT	WGS initiated
		c.14583−20C>G	p.(=)			
W19-0124	arRP	c.2299del	p.(Glu767Serfs*21)	Pathogenic	NT	WGS initiated
W19-0125	arRP	c.2276G>T	p.(Cys759Phe)	Pathogenic	NT	
		c.5516T>A	p.(Val1839Glu)	VUS*		
W19-0126	arRP	c.2299del	p.(Glu767Serfs*21)	Pathogenic	NT	WGS initiated
W19-0128	arRP	c.11864G>A	p.(Trp3955*)	Pathogenic	NT	
		c.9258+1G>A	p.(?)	Pathogenic		
W19-0855	USH2	c.11864G>A	p.(Trp3955*)	Pathogenic	Yes	
		c.4627+25435_4987+660del	p.(Gly1543_Pro1662del)	Pathogenic		
W19-0856	USH2	c.11864G>A	p.(Trp3955*)	Pathogenic	Yes	
		c.11048−2A>G	p.(?)	Pathogenic		
W19-0857	USH2	c.11864G>A	p.(Trp3955*)	Pathogenic	Yes	
		**c.8559−?_8681+?del**	p.(Tyr2854_Arg2894del)	Pathogenic		
W19-0858	USH2	c.11864G>A	p.(Trp3955*)	Pathogenic	Yes	
		**c.9388T>G**	p.(Trp3130Gly)	Likely Pathogenic		
W19-0859	USH2	c.11864G>A	p.(Trp3955*)	Pathogenic	Yes	
		c.12234_12235del	p.(Asn4079Trpfs*19)	Pathogenic		
W19-0860	USH2	c.11864G>A	p.(Trp3955*)	Pathogenic	Yes	
		c.6967C>T	p.(Arg2323*)	Pathogenic		
W19-0861	USH2	c.11864G>A	p.(Trp3955*)	Pathogenic	Yes	

		**c.14303A>C**	p.(Tyr4768Ser)	Likely Pathogenic		
		c.11864G>A	p.(Trp3955*)	Pathogenic		
W19-0862	USH2	c.1644+10004_1972−12164del	p.(Cys549_Gln657del)	Pathogenic	NT	
		c.11864G>A	p.(Trp3955*)	Pathogenic		
W19-0863	USH2	c.11328T>A	p.(Tyr3776*)	Pathogenic	Yes	
		c.11864G>A	p.(Trp3955*)	Pathogenic		
W19-0864	USH2	c.10712C>T	p.(Thr3571Met)	Likely Pathogenic	Yes	
		**c.11683G>T**	p.(Gly3895*)	Pathogenic		
W19-0921	USH2	c.9371+1G>C	p.(?)	Pathogenic	NT	
		**c.15286del**	p.(Glu5096Lysfs*6)	Pathogenic		
W19-0923	USH2	c.2299del	p.(Glu767Serfs*21)	Pathogenic	NT	
		c.9371+1G>C	p.(?)	Pathogenic		
W19-0924	USH2	c.7595−3C>G	p.(Pro2533Asnfs*5)	Pathogenic	NT	
		c.4645C>T	p.(Arg1549*)	Pathogenic		
W19-0943	USH2	c.1606T>A	p.(Cys536Ser)	VUS*	NT	
		c.8981G>A	p.(Trp2994*)	Pathogenic		
W19-0944	USH2	c.13010C>T	p.(Thr4337Met)	Likely Pathogenic	NT	
		c.8740C>T	p.(Arg2914*)	Pathogenic		
W19-0945	USH2	c.2299del	p.(Glu767Serfs*21)	Pathogenic	NT	
		c.8981G>A	p.(Trp2994*)	Pathogenic		
W19-0948	USH2	c.8740C>T	p.(Arg2914*)	Pathogenic	NT	
		c.2299del	p.(Glu767Serfs*21)	Pathogenic		
W19-0949	USH2	c.920_923dup	p.(His308Glnfs*16)	Pathogenic	NT	
		c.3187_3188del	p.(Gln1063Serfs*15)	Pathogenic		
W19-0950	USH2	c.8709C>T	p.(Arg2894_Asn2906del,=)	Pathogenic	NT	
		c.4510dup	p.(Arg1504Lysfs*26)	Pathogenic		
W19-0951	USH2				NT	WGS initiated

Variants that were reported as a first variant are underlined. Novel variants are indicated in bold. Classifications marked with an asterisk are VUS according to the ACMG guidelines but were predicted ‘potentially pathogenic’ as they met our pathogenicity criteria for missense variants. NT: not tested
