# Supplementary material for: Molecular Inversion Probe-Based Sequencing of USH2A Exons and Splice Sites as a Cost-Effective Screening Tool in USH2 and arRP Cases
Source: Int J Mol Sci. 2021 Jun 15;22(12):6419. doi: 10.3390/ijms22126419 (PMC8232728; doi:10.3390/ijms22126419)
Supplement: Supplementary file 1 [file ijms-22-06419-s001.zip › Supplementary data/Table S2.pdf]

Table S2: Sequences of primers employed to determine the CNV breakpoints that were identified in this study

CNV	Forward primer	Reverse primer
Exon 10-11 deletion	TCCTCAGAAAGCATGCAAGC	GGAGACACTACAACTGAAACCAC
Exon 22-24 deletion	AGTAGGGTAAGGACGAACAGAC	TGTGTGTGTGTGTTTTAGAGGG
Exon 38-56 deletion	AGCTCTATTCTGTTCCTGGCT	ACTCAAGAATCCAACTGTTTGCT
