# Supplementary material for: Molecular Inversion Probe-Based Sequencing of USH2A Exons and Splice Sites as a Cost-Effective Screening Tool in USH2 and arRP Cases
Source: Int J Mol Sci. 2021 Jun 15;22(12):6419. doi: 10.3390/ijms22126419 (PMC8232728; doi:10.3390/ijms22126419)
Supplement: Supplementary file 1 [file ijms-22-06419-s001.zip › Supplementary data/Table S3.pdf]

Table S3: Sequences of primers that were employed to create the constructs for the minigene splice assays.

Variant	Primers	Size of insert (nt)
c.8709C>T	GGGGACAAGTTTGTACAAAAAAGCAGGCTTCccatgccacaccacctttg	2278
	GGGGACCACTTTGTACAAGAAAGCTGGGTGgctgtaaccaaattcaaggctg	
c.14583-20C>G	GGGGACAAGTTTGTACAAAAAAGCAGGCTTCcgttaacacgtttgaggcac	4028
	GGGGACCACTTTGTACAAGAAAGCTGGGTGgccacgggaaatgcaaatac	

Sequence of the Gateway® *att*B site is depicted in upper case, the *USH2A*-specific sequence in lower case letters.
